# Basilar impression as complication of Grisel's syndrome

**DOI:** 10.1002/ccr3.1286

**Published:** 2017-12-08

**Authors:** Lasse Dührsen, Tammam Abboud, Lennart Viezens, Sven Oliver Eicker, Marc Dreimann

**Affiliations:** ^1^ Department of Neurosurgery University Medical Center Hamburg‐Eppendorf Hamburg Germany; ^2^ Department of Trauma, Orthopedic and Plastic Surgery University Medical Center Göttingen Göttingen Germany; ^3^ Department of Trauma, Hand and Reconstructive Surgery University Medical Center Hamburg‐Eppendorf Hamburg Germany

**Keywords:** Atlantoaxial joint, basilar impression, craniocervical junction, Grisel's syndrome

## Abstract

Grisel's syndrome presents a rare disease. Here, we present a peculiar case of Grisel's syndrome with an unfavorable course developing a basilar impression. This highlights the importance of close clinical and radiological follow‐up even in cases where the course seems uncomplicated.

## Introduction

Grisel's syndrome is a rare disease that is accompanied by a subluxation of the atlantoaxial joint 1. It occurs as a result of infection of the upper respiratory tract or after surgery of the head and neck area and frequently occurs, due to lax ligaments, in patients with trisomy 21 2. There are four stages according to the classification by Fieldings and Hawkins 3. Therapeutic options are generally immobilization with a soft collar and antibiotic treatment. Surgical therapy is reserved for severe and refractory cases. Here, we present a peculiar course of a patient with Grisel's syndrome who required urgent surgery due to progressive basilar impression.

## Case History

A 24‐year‐old man with trisomy 21 presented with neck pain (VAS 6/10) and the clinical picture of a Torticollis at our institution. Four weeks previously, he suffered an infection of the upper respiratory tract. In the suspicion of Grisel's syndrome, imaging with CT and MRI was performed. Here, the suspicion was confirmed and showed a C1–C2 subluxation, type III according to Fielding, and the onset of a subluxation of C0–C1 was seen 3. Antibiotic therapy had already taken place, and the laboratory signs of inflammation were back to normal. Closed reduction and fixation were unsuccessful. Therefore, immobilization in a cervical orthosis and close clinical and radiological controls were carried out. Four months later, a control CT scan of the craniovertebral junction revealed an increase in subluxation of C1 on C2 with increasing basilar impression. Clinically, there was a progressive rotatory fixation which could not be redressed to neutral position. The C1–C2 subluxation altered into a C1–C2 luxation. Neurological deficits were still absent at this point of time; however, the indication for surgery and stabilization was given in this unstable situation with distinctive pain syndrome and increasing torticollis in order to prevent neurological deterioration and anchorage of the rotatory fixation.

## Surgical Procedure

**Figure 1:**
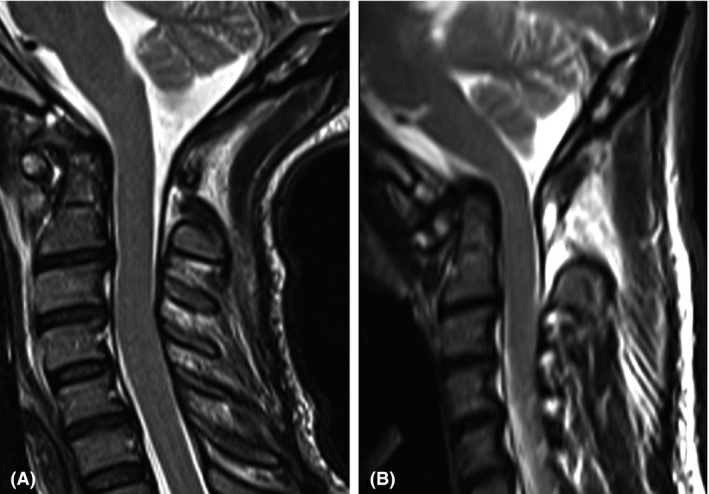
T2‐weighted sagittal MRI scans. In the early phase of the disease, only the luxation of C1–C2 seems relevant (A). The follow‐up MRI reveals basilar impression (B).

**Figure 2:**
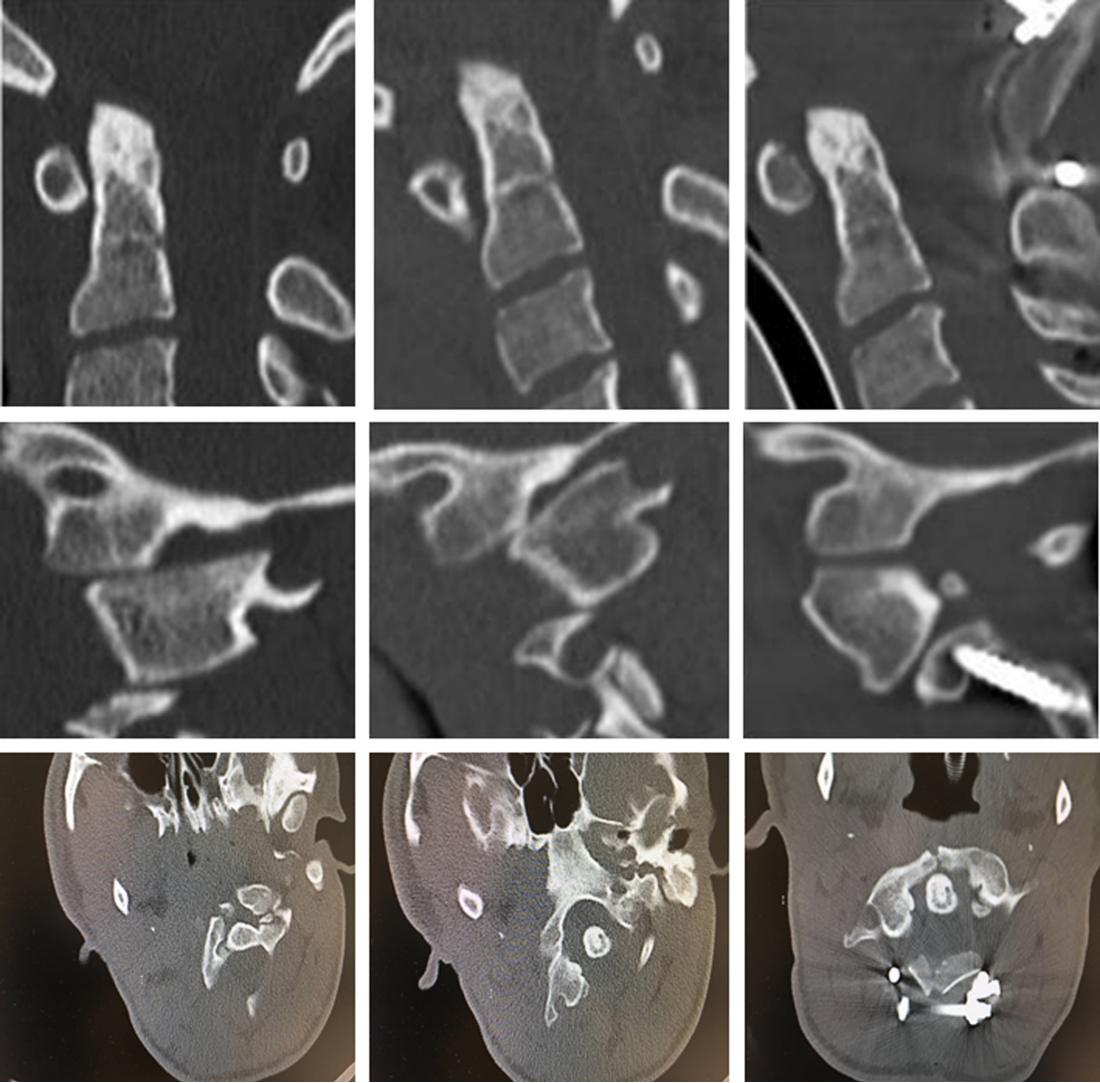
The importance of close radiological follow‐up is illustrated here. The sagittal and transversal CT scan showed an increase in C0/C1 subluxation over a period of 4 months (column 1 and 2). The postoperative sagittal CT scan confirmed successful dorsal decompression and reconstruction of the sagittal alignment (column 3).

**Figure 3:**
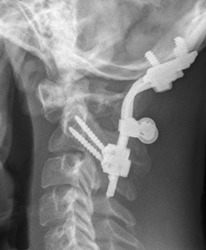
Two years postoperatively, plain X‐ray shows unchanged position of material and no evidence for resubluxation.

An open reduction and fusion with instrumentation of C0–C2 and extension of the foramen magnum were carried out. The patient was in prone position, and the head was fixated in a Mayfield. Closed reduction showed to be insufficient. Therefore, intraoperatively, the anatomical landmarks for instrumentation of C1 were difficult to identify. Fluoroscopy was of no major help due to rotation. It was decided to place isthmus screws in C2 and fix these to C0 (Synapse, Synthes) to prevent damage to Aa. vertebralis and spinal cord. Foramen magnum was widened, and we performed a laminectomy of C1. Spondylodesis was finished by inserting a pelvic bone from C0 to C2. The postoperative course was inconspicuous. The postoperative CT scan showed the reconstruction of the sagittal alignment and a correct position of implanted material and reposition of C0–C2 with regularly angulation in all planes (Figs 1, 2, 3).

## Radiographic and Clinical Follow‐up

In the two‐year postoperative control, the patient was free of symptoms; especially he did not complain about pain. A plain X‐ray of the craniocervical junction showed no evidence for resubluxation. There was no sign of torticollis left.

## Discussion

To our knowledge, this is the first case reporting of a Grisel's syndrome with the subsequent development of a C0–C1 instability with basilar impression and one of only fifteen cases concerning adults 4. Surgical intervention became necessary due to ineffective conservative therapy and the severe progressive basilar impression with clinical rotation and inclination of the neck and progressive pain. The patient showed a good clinical result afterward and on two‐year follow‐up visit.

Grisel's syndrome is a rare entity and was first described in 1930 1. The usual course shows symptoms of a torticollis days to weeks after an infection or a surgical intervention in the ear, nose and throat area. Trisomy 21 is a risk factor for Grisel's syndrome 5. The underlying pathomechanism is properly due to preexisting loose ligaments and the induction of spasm in the cervical muscles caused by inflammation 6. Grisel syndrome is classified by Fielding and Hawkins in four types 3. In all types, an antibiotic therapy should be admitted to treat the underlying cause. In addition, in type I and II, conservative treatment with immobilization in a cervical collar is recommended. In type III, the closed reduction is performed by means of halofixture, and in type IV, open reduction and fusion of C1–C2 should be performed 7. Most cases gain full recovery after such treatment. Basilar impression is generally associated with Chiari malformation, syringomyelia, and hydrocephalus 8. There are no reports concerning basilar impression following Grisel's syndrome.

In our case, conservative treatment was ineffective and subluxation was progressive with the complication of basilar impression. We therefore decided to perform an open reduction and surgical instrumentation of C1–C2 which is the generally accepted approach 4. Due to unrecognizable rotation and missing landmarks of C1, instrumentation had to be extended to C0. There is also evidence for an early surgical intervention especially in adults 4.

In conclusion, once the diagnosis of Grisel's syndrome has been put, a close clinical and radiological follow‐up is mandatory. The staged treatment according to the classification by Fielding should be applied in “classical” courses. The current literature recommends the generous indication for applying a Philadelphia soft collar which eventually can prevent a more serious surgical intervention 9. A treatment delay should therefore be avoided if possible. Surgery usually becomes inevitable when neurological deficits are imminent.

## Authorship

LD, TA, and SOE: summarized case information and drafted the manuscript. LV, SOE, and MD: performed surgery and participated in patient care in the hospital. MD: was responsible for follow‐up. All authors read and approved the final manuscript.

## Conflict of Interest

None declared.
